# Respiratory symptoms, sleep-disordered breathing and biomarkers in nocturnal gastroesophageal reflux

**DOI:** 10.1186/s12931-016-0431-7

**Published:** 2016-09-20

**Authors:** Össur Ingi Emilsson, Bryndís Benediktsdóttir, Ísleifur Ólafsson, Elizabeth Cook, Sigurður Júlíusson, Einar Stefán Björnsson, Sunna Guðlaugsdóttir, Anna Soffía Guðmundsdóttir, Ekaterina Mirgorodskaya, Evert Ljungström, Erna Sif Arnardóttir, Þórarinn Gíslason, Christer Janson, Anna-Carin Olin

**Affiliations:** 1 Faculty of Medicine, University of Iceland, Vatnsmyrarvegur 16, 101, Reykjavik, Iceland; 2 Department of Respiratory Medicine and Sleep, Landspitali University Hospital, Reykjavik, Iceland; 3 Department of Respiratory, Allergy and Sleep Research, Uppsala University, Uppsala, Sweden; 4 Department of Clinical Biochemistry, Landspitali University Hospital, Reykjavik, Iceland; 5 Department of Otolaryngology, Landspitali University Hospital, Reykjavik, Iceland; 6 Department of Gastroenterology, Landspitali University Hospital, Reykjavik, Iceland; 7 Department of Occupational and Environmental Medicine, University of Gothenburg, Gothenburg, Sweden; 8 Department of Chemistry and Molecular Biology, University of Gothenburg, Gothenburg, Sweden

**Keywords:** Nocturnal gastroesophageal reflux, Asthma, Bronchitis, Sleep-disordered breathing, Exhaled breath condensate, Particles in exhaled air

## Abstract

**Background:**

Nocturnal gastroesophageal reflux (nGER) is associated with respiratory symptoms and sleep-disordered breathing (SDB), but the pathogenesis is unclear. We aimed to investigate the association between nGER and respiratory symptoms, exacerbations of respiratory symptoms, SDB and airway inflammation.

**Methods:**

Participants in the European Community Respiratory Health Survey III in Iceland with nGER symptoms (*n* = 48) and age and gender matched controls (*n* = 42) were studied by questionnaires, exhaled breath condensate (EBC), particles in exhaled air (PEx) measurements, and a home polygraphic study. An exacerbation of respiratory symptoms was defined as an episode of markedly worse respiratory symptoms in the previous 12 months.

**Results:**

Asthma and bronchitis symptoms were more common among nGER subjects than controls (54 % vs 29 %, *p* = 0.01; and 60 % vs 26 %, *p* < 0.01, respectively), as were exacerbations of respiratory symptoms (19 % vs 5 %, *p* = 0.04). Objectively measured snoring was more common among subjects with nGER than controls (snores per hour of sleep, median (IQR): 177 (79–281) vs 67 (32–182), *p* = 0.004). Pepsin (2.5 ng/ml (0.8–5.8) vs 0.8 ng/ml (0.8–3.6), *p* = 0.03), substance P (741 pg/ml (626–821) vs 623 pg/ml (562–676), *p* < 0.001) and 8-isoprostane (3.0 pg/ml (2.7–3.9) vs 2.6 pg/ml (2.2–2.9), *p* = 0.002) in EBC were higher among nGER subjects than controls. Albumin and surfactant protein A in PEx were lower among nGER subjects. These findings were independent of BMI.

**Conclusion:**

In a general population sample, nGER is associated with symptoms of asthma and bronchitis, as well as exacerbations of respiratory symptoms. Also, nGER is associated with increased respiratory effort during sleep. Biomarker measurements in EBC, PEx and serum indicate that micro-aspiration and neurogenic inflammation are plausible mechanisms.

## Background

Gastroesophageal reflux (GER), and especially nocturnal GER (nGER), is associated with many symptoms from the respiratory tract, often asthma symptoms [1–3]. However, it is not fully known how GER patients with asthma symptoms differ from other asthma patients, and what the pathogenic mechanisms are.

Sleep-disordered breathing (SDB) and nGER are also closely linked. The strain on the gastroesophageal junction by increased respiratory effort in SDB is hypothesized to lead to nGER [4]. Conversely, persistent nGER seems to be a risk factor for developing symptoms of SDB [2]. However, obesity is a significant confounder as it increases the risk of both SDB and nGER [5].

Two different pathophysiological mechanisms have been proposed for the GER-induced respiratory symptoms and diseases. One involves micro-aspiration of gastric fluids into the lungs causing irritation and inflammation, while the second involves bronchoconstriction caused by a vagal reflex induced by acidic reflux to the distal oesophagus [6]. Studies on respiratory biomarkers in GER suggest these two mechanisms are probably associated with different extra-oesophageal manifestations [7]. For instance, pepsin has been described as a marker of aspiration, and neuroinflammatory markers such as neurokinin A and substance P associated with a vagal reflex [8, 9]. Further studies on the pathogenesis will increase our understanding of the interactions between nGER, SDB and respiratory symptoms, which will ultimately lead to a more appropriate treatment of these patients.

Our aim was to comprehensively investigate the association between nGER and respiratory symptoms, exacerbations of respiratory symptoms, lung function and SDB. We used various exhaled biomarkers to study if the two above mentioned mechanisms explained the association between nGER and respiratory disorders, and if the two mechanisms had different symptom profiles. We also hypothesized that SDB might affect the association between nGER and respiratory disorders.

## Methods

This study is based on a 20 years prospective, population-based cohort study in Reykjavik, Iceland, the European Community Respiratory Health Survey (ECRHS, see http://www.ecrhs.org). The study participants in ECRHS in Iceland were first studied in 1990 [10], re-studied in 2000 [11], and for the third time in 2012, when the participants were aged 40–65 years. Among the 522 subjects contacted in Iceland for ECHRS III, a total of 455 participated, or 87 % of those invited [12]. They participated in a structured interview, answered questionnaires including the Epworth sleepiness scale, underwent spirometry, measurements of height and weight, gave blood samples, and underwent a home polygraphic study. All participants reported their medications, including proton pump inhibitors (PPI), and if they used them on a regular basis or as needed. A subgroup also underwent a 24 h oesophageal pH-impedance measurement, the results of which have been reported elsewhere [13].

Of the 455 participants in ECRHS III in Iceland, 82 had symptoms suggestive of nGER. These 82 subjects were invited for a second visit in 2013, of which 71 (87 %) participated. Also, 63 age and gender paired controls from the ECRHS III cohort without any nGER symptoms were invited, of which 49 (78 %) participated. BMI pairing was attempted but only partially accomplished due to insufficient cohort size.

During the second visit all subjects participated in a structured medical interview, answered a modified reflux disease questionnaire [13], were weighted again, and examined with laryngoscopy. Exhaled breath condensate (EBC), particles in exhaled air (PEx) [14] and blood were sampled. Nitric oxide in exhaled air (FeNO) was measured. Height and the new weight were used to calculate body mass index (BMI).

As those with suspected nGER were invited first, the median time from first to second visit was 4.8 months for the nGER group and 8.4 months for the control group (*p* < 0.001).

The study was approved by the Bioethics Committee of Iceland (National Bioethics Committee of Iceland: VSN-11-121) and all participants signed informed consent.

### Structured medical interview

The interview was performed to collect information on health status changes from the last visit (2012). The interview asked specifically about changes in general health status and in medication, as well as recent respiratory and OSA symptoms using selected questions from the previous visit’s questionnaire; see further details in respective sections below.

In total, 7 cases and 7 controls reported changes in health status since their last visit. In the case group, health changes included diagnosis of haemochromatosis, removal of a liver cyst, lumbago etc. In the control group, health changes included coronary artery disease, discus prolapse, hypothyroidism, candida oesophagitis etc. Altogether 14 cases had any changes in their medical treatment, among which one discontinued PPI treatment, one started PPI treatment and 4 had changes in PPI treatment. Among controls, 6 had changes in their medical treatment, none of which regarded PPI. No use of other antacid medicine was reported.

### Definition of nGER

**Fig. 1:**
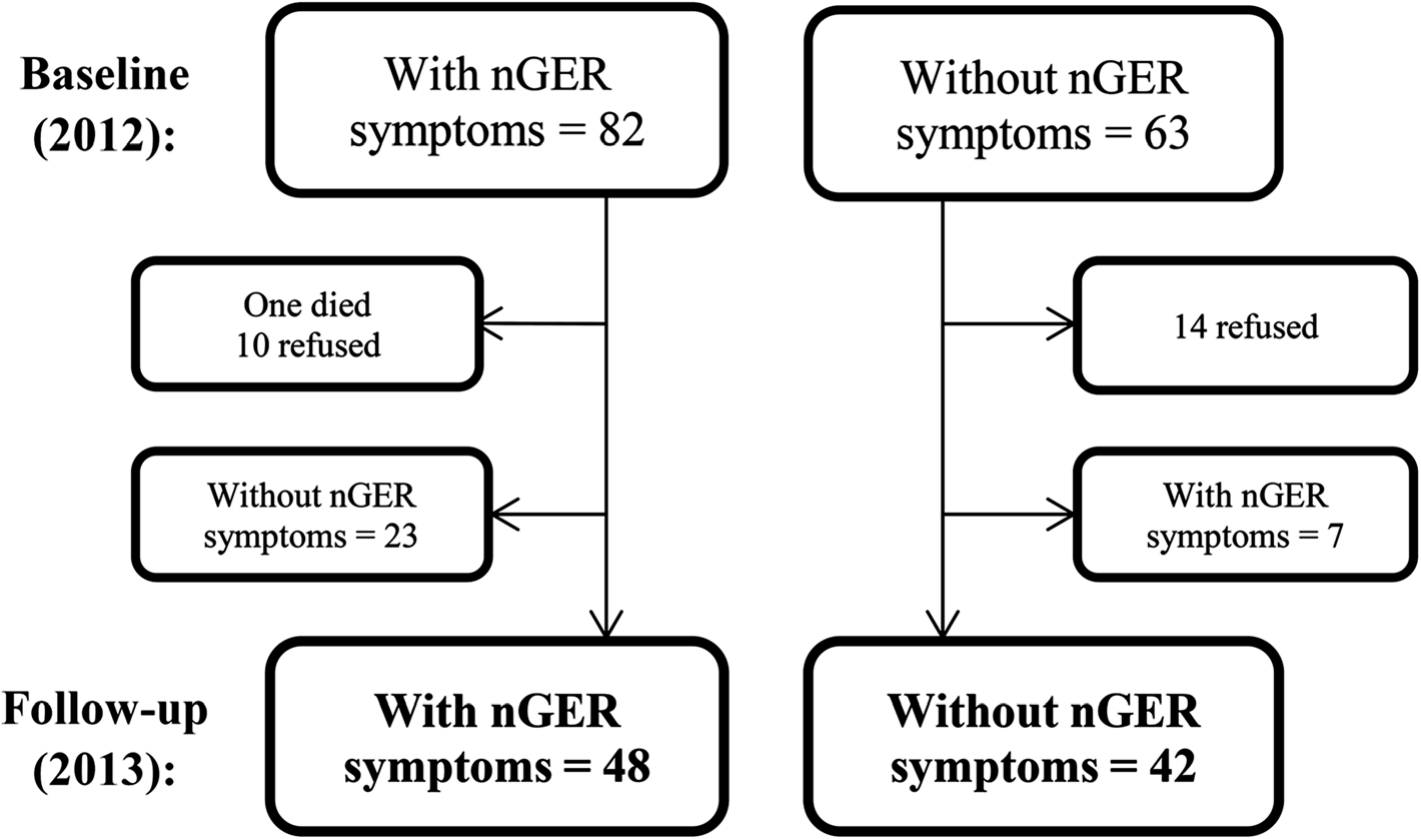
A flow diagram of selection of cases and controls. At the top are subjects at the time of first visit in the ECRHS III study, and at the bottom at follow-up around 5–8 months later. The groups in bold were used for further analysis: Subjects who were symptomatic at baseline and follow-up (persistent nGER), and subjects who were asymptomatic at baseline and follow-up (controls). nGER = nocturnal gastroesophageal reflux

The definition of nGER was based on a modified version of the Reflux Disease Questionnaire (RDQ) [13, 15]. The modification was that questions from the heartburn and regurgitation dimensions were posed specifically for daytime symptoms and nocturnal symptoms in the previous 4 weeks. The modified version has not been as thoroughly validated as the original RDQ, but has been shown to identify subjects with nGER sufficiently [13]. Those reporting any nocturnal GER symptoms on the first (2012) and second visit (2013) were defined as having nGER (*n* = 48). Those without nocturnal GER symptoms on the first and second visit were defined as controls (*n* = 42). Those with nocturnal GER symptoms on only one of the visits were excluded (Fig. 1).

### Respiratory and OSA symptoms

Participants were asked at follow-up about various respiratory symptoms in the previous 12 months, using questions from the main ECRHS III questionnaire (http://www.ecrhs.org) [10]. For analysis purposes, the symptoms were later grouped into asthma symptoms (wheeze, chest tightness, breathlessness at rest, breathlessness after exercise, nocturnal cough, nocturnal attacks of breathlessness) and bronchitis symptoms (cough or phlegm without ongoing respiratory infection). An exacerbation of respiratory symptoms was defined as a temporary marked increase in respiratory symptoms: cough, phlegm and shortness of breath. Frequent exacerbations were defined as at least two exacerbations in the previous 12 months. Current asthma was defined as questionnaire-reported physician diagnosed asthma as well as either experienced asthma symptoms in the previous 12 months and/or currently using any medicine for asthma [16].

Symptoms of OSA were analysed at follow-up with selected questions from a questionnaire developed by the Sleep Apnea Global Initiative Consortium (SAGIC) [12]. The participants were asked about sleep related symptoms in the previous month. Specifically, the questions regarded frequency of self-reported snoring and apneas on a five point frequency scale, and how sleepy they felt during the day on a five point scale (Not at all, a little bit, moderately, quite a bit, extremely). Snoring was considered positive if reported at least three times per week, and apneas if reported at least once per week. Daytime sleepiness was considered present if reported as moderate or more.

### Spirometry

Results from a pre- and post-bronchodilator spirometry done during the main ECRHS III study were used for assessment of airway obstruction and reversibility. Spirometry was performed in the same manner as in previous ECRHS studies [11]. Forced expiratory volume in one second (FEV1) and forced vital capacity (FVC) was recorded and reference values were calculated according to the NHANES III study [17]. Reversibility test was considered positive if FEV1 increased at least 200 ml after bronchodilation as well as increased at least 12 % compared to before bronchodilation.

### Home polygraphic studies

Subjects were invited for a home polygraphy with a T3 device (Nox Medical, Reykjavik, Iceland), a type 3 polygraphy as previously described [12]. The polygraphies were scored by two trained sleep technologists. Polygraphies were scored in accordance with the American Academy of Sleep Medicine 2007 manual using the accepted hypopnea classification requiring a ≥30 % drop in respiratory flow for ≥10 s with ≥4 % oxygen desaturation [18].

Snoring was measured using audio recordings from a sensor on the chest. Snore events were automatically scored using the Noxturnal 4.3 scoring algorithm, with an absolute threshold for snore events set as audio volume >65 dB. In comparison to manually scored snore events, this definition has been shown to have sensitivity and a positive predictive value of 0.79 and 0.94, respectively [19]. A snore index was calculated as the number of snores per hour of sleep.

### Collection of biosamples from exhaled air

EBC samples were collected with ECoScreen II (FILT - Lung- and Thorax Diagnostic GmbH, Germany) at -10 °C for 15 min. Participants wore a nose-clip and used tidal breathing. The samples collected were immediately divided into polypropylene test tubes (Screw cap micro tube 72.694.007, Sarstedt, Germany), 0.5 ml into each tube. Depending on the volume collected, the number of tubes varied from three to six. If any excess volume was left, it was measured and then disposed. 25 μl of a mixture of protease inhibitors was added to the first two test tubes (cOmplete, Mini version 10; Roche Diagnostics, Germany). Nothing was added to the third test tube. 5 μl of a 5 mg/ml solution of butylated hydroxytoluene in ethanol was added to the fourth test tube (Sigma-Aldrich, USA). If a fifth test tube was collected, 25 μl of the same protease inhibitors mixture was added as above. Other test tubes had no additives. The samples were immediately frozen at -20 °C, and within four hours moved to -80 °C for storage until measurements were performed.

FeNO was measured with NIOX MINO (Aerocrine, Sweden) according to manufacturer description (see http://www.niox.com). Participants were allowed a maximum of five repetitions to perform the measurement. However, more than one repetition was rarely needed and never more than three repetitions.

A method to collect particles in exhaled air has recently been established, using an instrument developed specifically for this purpose (PExA^TM^, Sahlgrenska University Hospital, Gothenburg, Sweden) [14]. The exhaled particles have been shown to reflect the respiratory tract lining fluid and to origin mainly from the small airways [20]. Before sample collection start, participants breathed filtered air for two minutes tidally to avoid contamination of ambient particles. A specific breathing manoeuvre, allowing for airway closure and re-opening to augment the number of exhaled particles, was applied. In this manoeuvre, the participants exhaled to residual volume, held their breath for three seconds, then inhaled sharply up to full inspiration, and finally exhaled with a velocity between 25 and 1000 ml/s to almost full expiration. Particles were only collected from the last exhalation of this manoeuvre. In between these breathing manoeuvres, the participants breathed filtered air tidally for 30–60 s. The breathing manoeuvre was repeated until 300 ng of PEx had been collected, or until the collection had taken 30 min in total, whichever came first.

The total mass of the collected particles was calculated based on the number and size of the particles, assuming them to be spherical and have a density of 1000 kg/m^3^ [21]. The exhaled particles were collected by impaction on a teflon filter (LCR Membrane Filter, Merck Millipore, Germany), which was divided into two halves immediately after sampling, and each half was stored in a polypropylene test tube (Screw cap micro tube, Sarstedt, Germany). The filters were immediately frozen at -20 °C, and within four hours moved to -80 °C until measurements were performed.

### Collection of plasma samples

Plasma samples were collected from a peripheral vein in EDTA-treated tubes. Albumin was measured directly, but for other measurements, the samples were frozen at -80 °C until measurements were performed.

### Biomarker measurements

Plasma albumin levels were measured using a Vitros 5.1 analyser and Vitros MicroSlide method (Ortho-Clinical Diagnostics, Rochester, USA). Albumin levels in EBC were determined by immunoturbidometry using a Virtos 5.1 analyser and reagents from Ortho-Clinical Diagnostics. Plasma high sensitivity C-reactive protein (hs-CRP) was measured on a Cobas 411 analyser using reagents from Roche Diagnostics, Mannheim, Germany. Interleukin 8 (IL-8) levels in plasma and EBC samples were determined using ELISA reagents (IBL International GmbH, Hamburg, Germany). Additionally, we performed ELISA measurements in EBC of pepsin (Wuhan EIAAB Science Co, Wuhan, China), 8-isoprostane (Cayman Chemical Company, Ann Arbor, MI, USA), neurokinin A (RayBiotech Inc, Norcross, A, USA) and substance P (Cayman Chemical Company, Ann Arbor, MI, USA). From the PEx samples, we registered data on exhaled particles’ number and mass, and measured albumin and surfactant protein A as markers of pulmonary inflammation as previously described [21]. Prior to protein assay of the PEx samples, the particles were extracted from Teflon filters using phosphate-buffered saline (PBS), containing 1 % bovine serum albumin (BSA), w/v, and 0.05 % Tween-20, v/v. 140 μl of the extraction buffer was added to each sample, followed up by 60 min shaking at 400 rpm and 37 °C at a thermomixer (Thermomixer comfort, Eppendorf; Eppendorf AG, Hamburg, Germany). Surfactant protein A (SP-A) in PEx and plasma was quantified using human SP-A ELISA kit (BioVendor, Czech Republic) and albumin using human albumin ELISA kit (Immunology Consultants Laboratory, Inc., USA), according to the manufacturer’s instructions, with small modifications. The human albumin ELISA assay has no cross-reactivity to bovine albumin.

When biomarkers were not measurable, because the concentration was below the lower limit of detection, their value was set as half the lower limit of detection for statistical analysis.

### Statistical analysis

All statistics were calculated with STATA 13.0 software, version intercooled (Stata Corporation, College Station, Texas). Associations and adjusted calculations were analysed as appropriate by chi square test, Fisher’s exact test, Wilcoxon rank-sum test, Spearman’s rank-order correlation and linear regression. A *p*-value of < 0.05 was considered statistically significant. As the aim was to give a comprehensive view of the complex association between nGER, respiratory symptoms and sleep-disordered breathing, no specific outcome variables were described as main outcomes.

Sensitivity analysis was done by adjusting for BMI as pairing was only partially accomplished. For parametric tests, a multiple linear regression was used. For categorical and non-parametric tests, calculations were redone with above mentioned tests, while either adjusting for BMI, or excluding those with BMI 35 or over when adjustment calculations were not applicable (one subject from control group and 7 subjects from nGER group excluded) (mean BMI ± SD: control group 26.9 ± 3.7; nGER group 27.6 ± 2.9, *p* = 0.38).

## Results

No difference was found between the two study groups in terms of age, gender, smoking status or incidence of arterial hypertension or diabetes (Table 1). The nGER group had a higher BMI, although statistically non-significant. Current asthma was significantly more common in the nGER group (27 % vs 7 %, *p* = 0.01).

**Table 1:** Subject characteristics

	No nGER (*n* = 42)	nGER (*n* = 48)	*p*-value
Age (mean ± SD)	56.4 ± 7.0	55.8 ± 6.7	0.70
Female gender	48 %	46 %	0.87
BMI (median (IQR))	25.9 (24.5–29.7)	28.1 (26.4–31.0)	0.07
Arterial hypertension	31 %	35 %	0.65
Diabetes	2.4 %	4.2 %	0.55
Ischaemic heart disease	2.4 %	4.2 %	0.55
Heart failure	0	2.1 %	0.53
Stroke	2.4 %	4.2 %	0.55
Smoking status:			
- Non-smoker	45 %	33 %	ref.
- Former smoker	45 %	46 %	0.49
- Current smoker	10 %	21 %	0.10
Current asthma	7.1 %	27.1 %	0.01
- On any asthma medication	7.1 %	12.5 %	0.49
Regular use of PPI	0	29 %	<0.01

*SD* standard deviation, *IQR* interquartile range, *PPI* Proton Pump Inhibitor, *ref* reference

### Respiratory symptoms and spirometry

**Fig. 2:**
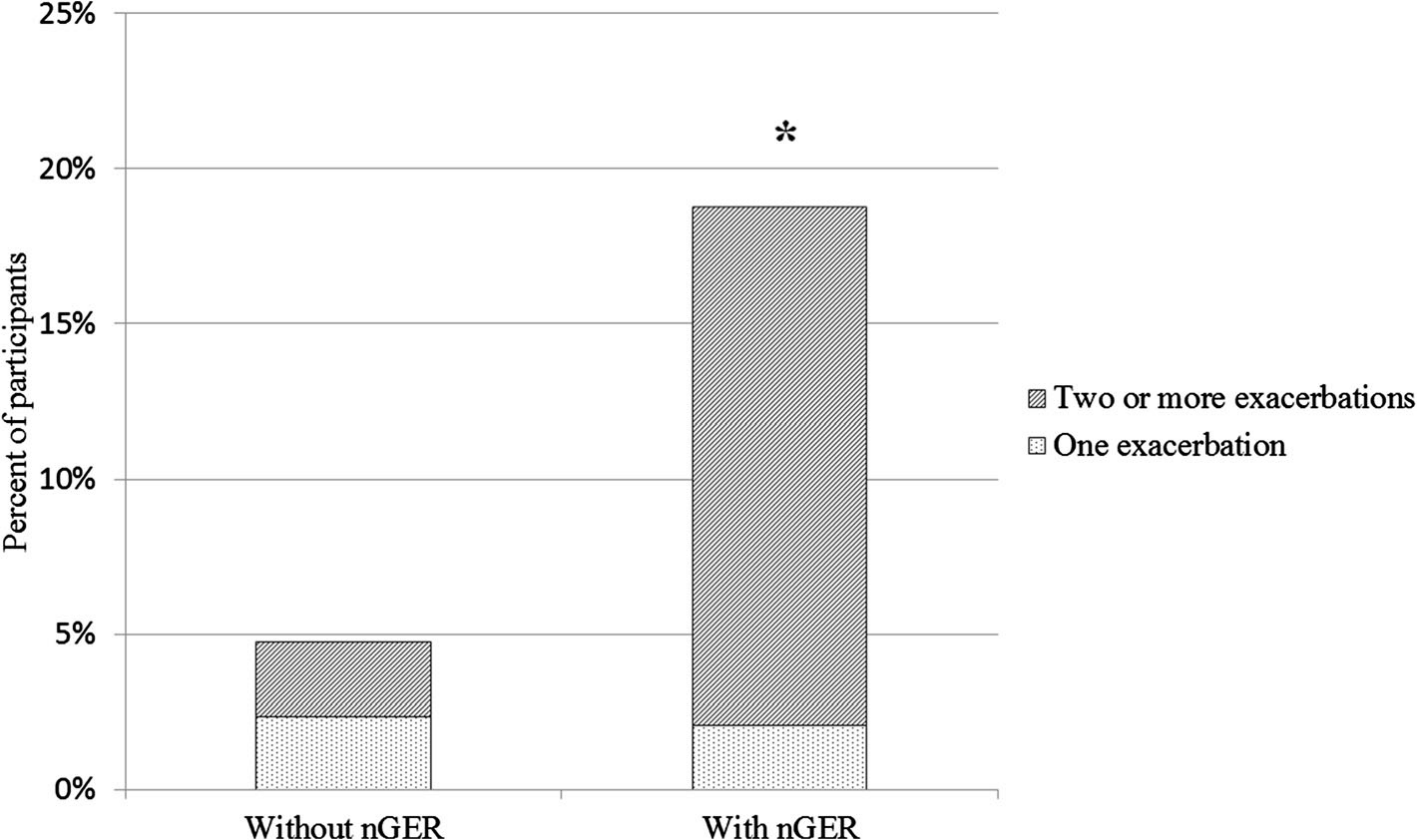
Exacerbations of respiratory symptoms in the previous 12 months among subjects with or without nGER. * *p* = 0.02 by chi square test

Symptoms of asthma and bronchitis were significantly more common among nGER subjects. Specifically, symptoms such as chest tightness, breathlessness, cough and phlegm were more common among nGER subjects (Table 2). Exacerbations of respiratory symptoms were significantly more common among nGER subjects. This difference was more pronounced when looking at frequent exacerbations (Fig. 2).

**Table 2:** Respiratory symptoms and nocturnal gastroesophageal reflux

	No nGER (*n* = 42)	nGER (*n* = 48)	*p*-value
Any asthma symptom:	28.6 %	54.2 %	0.01
Wheeze	16.7 %	27.1 %	0.24
- with breathlessness	4.8 %	14.6 %	0.12
- without cold	14.3 %	22.9 %	0.30
Chest tightness	0.0 %	14.6 %	0.01
Breathlessness at rest	0.0 %	14.6 %	0.01
Breathlessness after exercise	9.5 %	31.3 %	0.01
Nocturnal attacks of breathlessness	0.0 %	8.3 %	0.06
Nocturnal cough	14.3 %	29.2 %	0.09
Any bronchitis symptom:	26.2 %	60.4 %	<0.01
Chronic morning cough	9.5 %	29.2 %	0.02
Chronic cough	14.3 %	33.3 %	0.04
- Coughing at least 3mo/y	11.9 %	27.7 %	0.07
Chronic morning phlegm	19.1 %	39.6 %	0.03
Chronic phlegm	9.5 %	20.8 %	0.14
- Phlegm at least 3mo/y	18.0 %	37.8 %	0.05

No significant differences were observed in the post bronchodilator forced expiratory volume in one second (FEV1), forced vital capacity (FVC), FEV1/FVC or reversibility.

BMI was positively associated with more exacerbations of respiratory symptoms among those with nGER (*p* = 0.046). Other findings were not affected by adjusting for BMI.

### Sleep-disordered breathing

Three subjects in the control group had a previously diagnosed OSA, whereof two were currently on treatment with positive airway pressure. Two subjects in the nGER group had a previously diagnosed OSA, whereof one was currently on treatment with positive airway pressure.

Reported snoring was more common among subjects with nGER, as well as daytime sleepiness. Apnea-hypopnea index (AHI) was significantly higher among nGER subjects compared with controls, as well as audio measured snoring (Table 3).

**Table 3:** Symptoms and signs of obstructive sleep apnea and nocturnal gastroesophageal reflux

	No nGER (*n* = 42)	nGER (*n* = 48)	*p*-value
Self-reported symptoms:			
Snoring ≥3 x week	23.8 %	44.7 %	0.04
Witnessed apneas ≥1 x week	2.4 %	12.5 %	0.08
Daytime sleepiness	9.8 %	46.8 %	<0.001
Epworth sleepiness scale >10	22 %	39 %	0.08
Epworth sleepiness scale (mean ± SD)	6.6 ± 4.0	8.2 ± 5.4	0.12
Polygraphy results:			
Moderate/severe OSA	10.5 %	26.2 %	0.07
AHI (median (IQR))	1.9 (0.5–8.0)	4.8 (1.4–16.2)	0.03
Snore index (snores per hour of sleep) (median (IQR))	67 (32–182)	177 (79–281)	0.004
Snore groups:			0.01
Snore index ≤ 100	61 %	26 %	
Snore index 101–250	24 %	45 %	
Snore index > 250	16 %	29 %	

*SD* standard deviation, *OSA* obstructive sleep apnea, *AHI* apnea-hypopnea index, *IQR* interquartile range

**Fig. 3:**
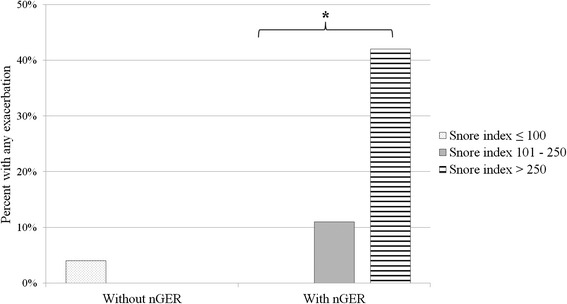
A positive association was found between snoring and exacerbations of respiratory symptoms among subjects with nGER, but not among subjects without nGER. * *p* = 0.03 by linear regression

Snoring was positively associated with exacerbations of respiratory symptoms among those with nGER, but not among those without nGER (Fig. 3). Furthermore, among those with nGER, snoring was only significantly associated with exacerbations among subjects with measurable pepsin in EBC (non-measurable pepsin in EBC: *p* = 0.33, measurable pepsin in EBC: *p* = 0.03).

After adjusting for BMI, AHI was no longer associated with nGER, but audio measured snoring was still significantly associated with nGER after adjusting for BMI. Other findings were not affected by adjusting for BMI.

### Biomarker analysis

Pepsin levels were higher in the nGER group. Substance P and 8-isoprostane were also higher in EBC among subjects with nGER than those without nGER. In PEx, both albumin and surfactant protein A (SP-A) were lower in subjects with nGER than in those without. In plasma samples, no difference was found in SP-A, hs-CRP or IL-8 concentrations between nGER subjects and controls (Table 4).

**Table 4:** Biomarkers in subject samples, measured in exhaled air, exhaled breath condensate, particles in exhaled air, and plasma

	No nGER (*n* = 42)	nGER (*n* = 48)	*p*-value
Exhaled air:			
FeNO (ppb)	15 (13–20)	17 (11–25)	0.48
EBC:			
Substance P (pg/ml)	623 (562–676)	741 (626–821)	<0.001
Neurokinin A (% detected)	17 %	30 %	0.13
Pepsin (ng/ml)	0.8 (0.8–3.6)	2.5 (0.8–5.8)	0.03
IL-8 EBC (% detected)	12 %	19 %	0.37
8-isoprostane (pg/ml)	2.6 (2.2–2.9)	3.0 (2.7–3.9)	0.002
PEx:			
Albumin (mg/g^a^)	73 (57–91)	48 (31–69)	<0.001
SP-A (mg/g^a^)	38 (31–43)	25 (20–35)	<0.001
Albumin/SP-A (ratio)	1.92 (1.50–2.64)	1.74 (1.26–2.95)	0.79
Plasma:			
hs-CRP (mg/l)	1.05 (0.68–2.26)	1.08 (0.62–1.94)	0.96
IL-8 plasma (% detected)	57 %	52 %	0.63
SP-A (ng/ml)	34.7 (29.8–48.6)	35.6 (27.6–47.0)	0.81

Values presented as “median (interquartile range)” unless otherwise stated

*SP-A* Surfactant protein A, *IL-8* Interleukin 8, *FeNO* Fraction of exhaled nitric oxide, *hs-CRP* High sensitivity C-reactive protein, *ppb* parts per billion

^a^Measured as mg of protein per gram of exhaled particles

**Fig. 4:**
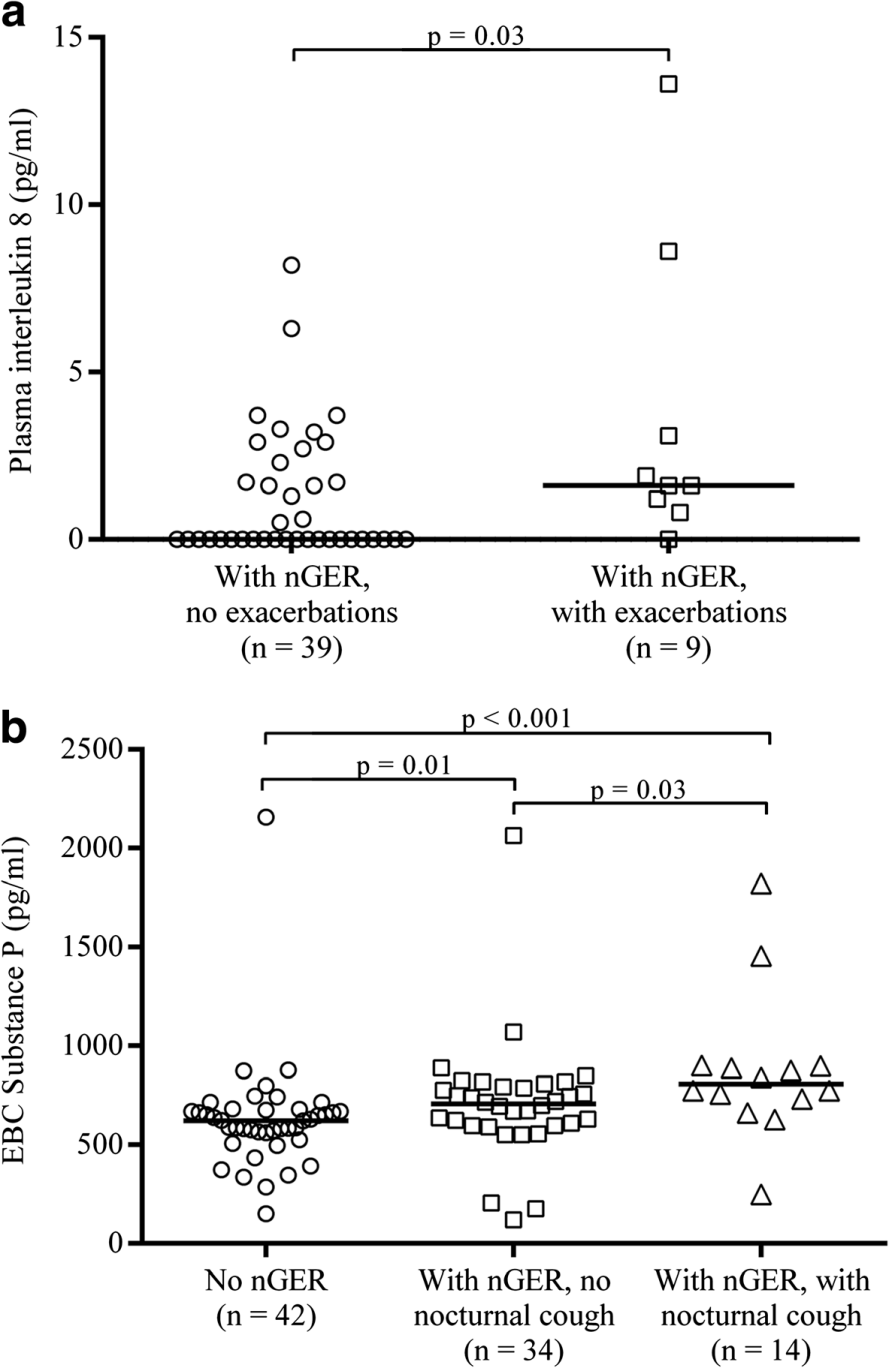
**a** Interleukin 8 levels in plasma among nGER subjects with or without exacerbations of respiratory symptoms. The transverse line represents median value. **b** Substance P levels in exhaled breath condensate among subjects with nGER with or without nocturnal cough. The transverse line represents median value. *P*-values calculated with Wilcoxon rank-sum test

In the nGER group, IL-8 in plasma was significantly higher among those with exacerbations of respiratory symptoms than in those without exacerbations (median (IQR): 1.6 pg/ml (1.2–3.1) vs. 0.2 pg/ml (0.2–2.3), *p* = 0.03) (Fig. 4a). In the nGER group, substance P was higher among those with nocturnal cough than those without nocturnal cough (708 pg/ml (597–793) vs. 808 pg/ml (732–900), *p* = 0.03) (Fig. 4b). No other significant differences were found in biomarkers in the nGER group in relation to exacerbations or respiratory symptoms.

The biomarker findings were not affected by adjusting for BMI.

## Discussion

In a general population sample, nGER was significantly associated with symptoms of asthma and bronchitis, as well as exacerbations of respiratory symptoms. Symptoms of SDB were also more common among those with nGER, and they had a higher number of measured events on a polygraphy. Participants with nGER had higher pepsin and higher inflammatory biomarkers in exhaled air. The biomarker findings support both theories on micro-aspiration of gastric fluids (increased pepsin and 8-isoprostane in EBC) and neurogenic inflammation through a vagal reflex (increased substance P in EBC) as causes of nGER related respiratory symptoms.

### Respiratory symptoms

The positive associations between nGER and respiratory symptoms as well as exacerbations of respiratory symptoms in the current study are in accordance with previous studies that have shown an association between GER and respiratory symptoms, COPD and asthma exacerbations [2, 22, 23]. Our study adds that the effect of nGER on the airways might be mediated by pepsin, 8-isoprostane and substance P. BMI had little effect on these results. Additionally, adjusting for both smoking and BMI did not significantly affect the results (data not shown).

We found IL-8 in plasma to be elevated among participants with both nGER and exacerbations. In asthmatic children, IL-8 has been found to be increased in bronchoalveolar lavage (BAL) only among those with GER [24]. Additionally, a correlation between IL-8 and bile acids in BAL has been demonstrated in lung transplanted patients who developed bronchiolitis obliterans syndrome [25]. In the current study, it is possible that the elevated IL-8 concentration was more related to frequent exacerbations in general, as these subjects were almost exclusively in the nGER group. However, as the previously mentioned studies found IL-8 to be increased specifically in GER in two different patient groups, we suspect this rather reflects the airway inflammation related to GER. This data therefore suggests a specific role of IL-8 in neutrophil recruitment in GER related respiratory conditions, related to micro-aspiration of gastric contents.

In our study, the oxidative stress biomarker 8-isoprostane was increased in EBC among those with nGER, as has previously been shown for GER in subjects with asthma [26]. This might reflect that persistent nGER leads to oxidative stress and inflammation of the airways, likely through direct effects of gastric contents on the airway epithelium.

We found significantly higher levels of the neuroinflammatory marker substance P in EBC among those with nGER, especially among those with nocturnal cough, suggestive of a neural mediated association between nGER and nocturnal cough. This finding is in accordance with previous studies on neuroinflammatory markers in GER and chronic cough [27]. Indeed, studies on distal oesophageal acid perfusion show a significant stimulus of the vagal nerve, with resulting bronchoconstriction and heart rate variation [28]. In support of the so-called reflex theory, we hypothesize that GER-induced vagal stimulation can cause neurogenic inflammation in the lungs, with cough being the main resulting symptom. Further studies are needed to evaluate this hypothesis.

Albumin and SP-A levels were lower in PEx among those with nGER, indicating an effect on small airways. The reduced proportion of surfactant protein A in the small airways lining fluid is likely to impact on surfactant function and host defence [29]. In turn, this might explain the association between nGER and exacerbations of respiratory symptoms. These findings suggest that nGER not only affects the proximal airways, but also the distal airways.

Of note, plasma hs-CRP was not associated with nGER, suggesting that the elevated inflammatory markers seen in EBC and PEx represented a local effect of nGER without a systemic effect.

### Sleep-disordered breathing

In the present study, nGER was associated with SDB and SDB-related symptoms. Previously, persistent nGER has been shown to increase the new onset of SDB symptoms, and SDB treatment with continuous positive airway pressure reduces nGER [2, 30]. SDB can likely lead to nGER through strain of the lower oesophageal sphincter, and nGER worsen SDB through increased proximal airway inflammation, resulting in a negative spiral [31, 32]. Of the objectively measured markers for SDB, we found the strongest association between nGER and audio measured snoring, especially after adjusting for BMI. This association has been reported but is much less studied than the association with AHI [33]. Interestingly, AHI has been found to correlate poorly with patient-centred outcomes such as quality of life [34]. Also, snoring has been associated with excessive daytime sleepiness and daytime fatigue independently of AHI [35].

We found that nGER and snoring had a synergistic effect in their association to exacerbations of respiratory symptoms. Previous studies have found such exacerbations to be associated with nGER, as well as SDB, but to our knowledge this synergistic effect has not been described before [22, 36]. As this effect was more pronounced among those with measurable pepsin in EBC, we hypothesize that SDB-associated nGER causes exacerbations of respiratory symptoms by micro-aspirations of gastric contents, but further research is needed.

### Strengths and limitations

The strengths of this study lie in the detailed analysis of respiratory biomarkers and home polygraphic studies performed in a well-defined study population based on a random general population sample. However, there were also certain limitations to this study. First, given the background of the study population, the number of severely symptomatic cases was low, resulting in a generally mild disease burden. Consequently, biomarkers may have been less affected than in a group with more severe disease, increasing the risk for false negatives. Second, biosamples were collected at different time points during the day, and therefore the biomarker measurements could be affected by circadian variability. However, as participants’ visits were similarly spread over time of day, circadian variability was unlikely to affect our results. Third, there is some uncertainty regarding where the biomarkers measured in EBC are derived from, and the water-vapour dilution of these samples is difficult to adjust for. Additionally, many of the biomarkers in EBC were near or below the lower limit of detection, making the measurements somewhat unreliable. However, the biomarkers which were well above the detection limit in EBC such as pepsin, substance P and 8-isoprostane differed clearly between the nGER and control group.

## Conclusions

We found that nocturnal gastroesophageal reflux associates with asthma and bronchitis symptoms as well as exacerbations of respiratory symptoms. The association is further supported by significant changes in airway inflammatory biomarkers, both in large and small airways. On the other hand, those with nocturnal gastroesophageal reflux and nocturnal cough had mostly increased signs of neurogenic inflammation. This indicated that the two hypothesized mechanisms of micro-aspiration and neurogenic inflammation might both be true, but associated with somewhat different symptoms. Snoring was also associated with nocturnal gastroesophageal reflux, and when combined with nocturnal gastroesophageal reflux, it strengthened the association with exacerbations of respiratory symptoms. This association between nocturnal gastroesophageal reflux and respiratory symptoms, where micro-aspiration, neurogenic inflammation and sleep-disordered breathing seem to be the responsible mechanisms, needs to be studied further.

## Abbreviations

AHI: Apnea-hypopnea index
BAL: Bronchoalveolar lavage
BMI: Body mass index
BSA: Bovine serum albumin
EBC: Exhaled breath condensate
ECRHS: European Community Respiratory Health Survey
ELISA: Enzyme-linked immunosorbent assay
FeNO: Fraction of exhaled nitric oxide
FEV1: Forced expiratory volume in one second
FVC: Forced vital capacity
GER: Gastroesophageal reflux
hs-CRP: High sensitivity C-reactive protein
IL-8: Interleukin 8
IQR: Interquartile range
mo/y: Months/year
nGER: Nocturnal gastroesophageal reflux
NHANES: National Health and Nutrition Examination Survey
OSA: Obstructive sleep apnea
PBS: Phosphate-buffered saline
PEx: Particles in exhaled air
ppb: Parts per billion
PPI: Proton pump inhibitor
RDQ: Reflux Disease Questionnaire
ref.: Reference
SAGIC: Sleep Apnea Global Initiative Consortium
SD: Standard deviation
SDB: Sleep-disordered breathing
SP-A: Surfactant protein A
