# Erratum to: Respiratory symptoms, sleep-disordered breathing and biomarkers in nocturnal gastroesophageal reflux

**DOI:** 10.1186/s12931-016-0448-y

**Published:** 2016-10-18

**Authors:** Össur Ingi Emilsson, Bryndís Benediktsdóttir, Ísleifur Ólafsson, Elizabeth Cook, Sigurður Júlíusson, Einar Stefán Björnsson, Sunna Guðlaugsdóttir, Anna Soffía Guðmundsdóttir, Ekaterina Mirgorodskaya, Evert Ljungström, Erna Sif Arnardóttir, Þórarinn Gíslason, Christer Janson, Anna-Carin Olin

**Affiliations:** 1 Faculty of Medicine, University of Iceland, Vatnsmyrarvegur 16, 101, Reykjavik, Iceland; 2 Department of Respiratory Medicine and Sleep, Landspitali University Hospital, Reykjavik, Iceland; 3 Department of Respiratory, Allergy and Sleep Research, Uppsala University, Uppsala, Sweden; 4 Department of Clinical Biochemistry, Landspitali University Hospital, Reykjavik, Iceland; 5 Department of Otolaryngology, Landspitali University Hospital, Reykjavik, Iceland; 6 Department of Gastroenterology, Landspitali University Hospital, Reykjavik, Iceland; 7 Department of Occupational and Environmental Medicine, University of Gothenburg, Gothenburg, Sweden; 8 Department of Chemistry and Molecular Biology, University of Gothenburg, Gothenburg, Sweden

## Erratum

In the original publication of this article [1], one of the co-author names was listed incorrectly. Þórarinn Gíslason should therefore have been written as Thórarinn Gislason.
